# Functional Source Separation-Identified Epileptic Network: Analysis Pipeline

**DOI:** 10.3390/brainsci12091179

**Published:** 2022-09-01

**Authors:** Elzbieta Olejarczyk, Filippo Zappasodi, Lorenzo Ricci, Annalisa Pascarella, Giovanni Pellegrino, Luca Paulon, Giovanni Assenza, Franca Tecchio

**Affiliations:** 1 Nalecz Institute of Biocybernetics and Biomedical Engineering, Polish Academy of Sciences, 02-109 Warsaw, Poland; 2 Department of Neuroscience, Imaging and Clinical Sciences, & Institute for Advanced Biomedical Technologies, ‘G. d’Annunzio’ University, 66100 Chieti, Italy; 3 Unit of Neurology, Neurophysiology, Neurobiology, Department of Medicine, Università Campus Bio-Medico di Roma, 00128 Rome, Italy; 4 Institute for Applied Mathematics “Mauro Picone” (IAC), National Research Council, 00185 Rome, Italy; 5 IRCCS San Camillo Hospital, 30126 Venice, Italy; 6 Laboratory of Electrophysiology for Translational Neuroscience, Institute of Cognitive Sciences and Technologies, National Research Council, 00185 Rome, Italy; 7 Faculty of Psychology, UTIU Uninettuno University, 00186 Rome, Italy

**Keywords:** focal epilepsy, EEG, transcranial Direct Current Stimulation (tDCS), Functional Source Separation (FSS), Higuchi Fractal Dimension (HFD), Directed Transfer Function (DTF)

## Abstract

This proof-of-concept (PoC) study presents a pipeline made by two blocks: 1. the identification of the network that generates interictal epileptic activity; and 2. the study of the time course of the electrical activity that it generates, called neurodynamics, and the study of its functional connectivity to the other parts of the brain. Network identification is achieved with the Functional Source Separation (FSS) algorithm applied to electroencephalographic (EEG) recordings, the neurodynamics quantified through signal complexity with the Higuchi Fractal Dimension (HFD), and functional connectivity with the Directed Transfer Function (DTF). This PoC is enhanced by the data collected before and after neuromodulation via transcranial Direct Current Stimulation (tDCS, both Real and Sham) in a single drug-resistant epileptic person. We observed that the signal complexity of the epileptogenic network, reduced in the pre-Real, pre-Sham, and post-Sham, reached the level of the rest of the brain post-Real tDCS. DTF changes post-Real tDCS were maintained after one month. The proposed approach can represent a valuable tool to enhance understanding of the relationship between brain neurodynamics characteristics, the effects of non-invasive brain stimulation, and epileptic symptoms.

## 1. Introduction

Recently, we started observing results indicating that cortical areas can be characterized by the temporal evolution of the electrical activity of local neuronal pools, the neurodynamics, dictated concurrently by their structure and networking within the whole brain [1,2,3,4,5,6,7].

Epilepsy is a neurological disease that affects about 1% (65 million people) of the worldwide population and is characterized by epileptic seizures, i.e., events that can vary from brief and nearly undetectable lapses to longer-lasting bouts of vigorous shaking [8,9]. Epilepsy is sustained by an excessive neuronal synchronization and altered functioning of the inhibition-excitation networks [10]. Medications control seizures in about 70% of cases [11]. When seizures do not respond to medications (Drug Resistant Epilepsy–DRE–people) surgery or neuromodulation may be considered [12]. Neuromodulation is the variation in the target’s excitability, changing its relationship with other brain regions and thus its functional behavior, which invasive (deep brain stimulation, DBS or vagal nerve stimulation, VNS) or non-invasive (repetitive transcranial magnetic stimulation, rTMS or transcranial Electrical Stimulation -tES) techniques can achieve. Neuromodulation via VNS and DBS are effective non-surgical treatments for DRE [13], but are both invasive techniques. Recently, non-invasive tES induced by low intensity currents delivered through electrodes placed on the subject’s scalp [14,15,16] started to be considered against DRE [17,18,19].

Following the promise of exploiting the neurodynamics of the target network to enhance the tES efficacy [3,20], the present work is a proof-of-concept (PoC) study aimed at exploring the methodological capability of the Functional Source Separation (FSS) [21] algorithm to identify the activity of the epileptogenic network, enabling you to monitor its neurodynamics in multiple conditions of interest. Significantly, the FSS was used to describe the activity of the remaining part of the brain, in order to access its neurodynamics and functional connectivity with the epileptogenic network. As an example of using the analysis pipeline, we studied the changes in brain network activity in a person with DRE who underwent Sham and Real transcranial direct current stimulation (tDCS), which improved clinical symptoms [17,22]. For this person we identified the two networks, the one that generates interictal activity and the one that describes the remaining part of the brain, and studied their neurodynamics as derived by a continuous resting electroencephalogram (EEG) before and after Sham and Real tDCS through a complexity measure. Furthermore, under the same four conditions, we assessed the functional connectivity between the two networks.

## 2. Materials and Methods

### 2.1. FSS Analysis Pipeline

#### 2.1.1. Network Identification via Functional Source Separation (FSS)

**Figure 1:**
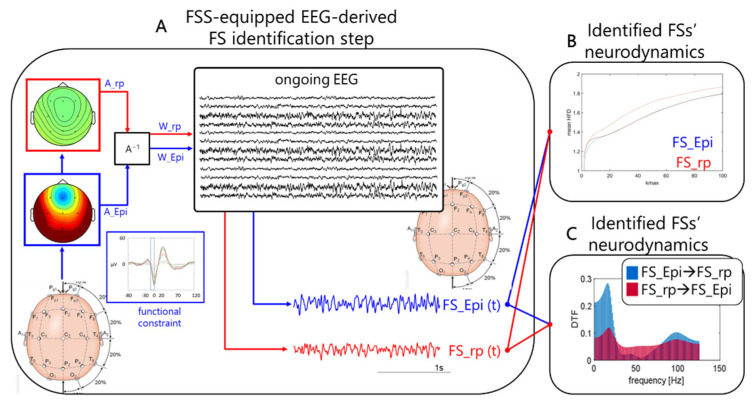
FSS identification step. Obtaining individual FS_Epi and FS_rp neurodynamics in resting states of interest. (**A**) Schematic depiction of how Functional Source Separation (FSS) obtains the functional source describing the epileptogenic network for individual patient and gives access to its activity while he/she was resting with closed eyes (Ongoing electroencephalographic (EEG)). FSS received the EEG data recorded along a sufficient time period for the interictal activity occurring tens of times to identify the SW patterns (functional constrain inset, with the 4 ms time window highlighted) as input and provided the A_Epi as an outcome (which, similar to W_Epi, is a time invariant 19-dimension vector). The FS_Epi scalp topography derived from A_Epi is shown. FS_Epi(t) in the four conditions of interest (before and after Real and Sham transcranial Direct Current Stimulation (tDCS)) (bottom right traces) is obtained through W_Epi multiplied with the EEG recording at rest in the corresponding condition (central inset). Similarly for FS_rp. From the time evolution of the two sources, we estimate their Higuchi Fractal Dimension (HFD) as a function of Kmax (**B**), and the directed transfer function (DTF) from each of the two on the other (**C**). A^−1^. FS, functional source.

The first step of the pipeline requires the EEG-derived selection of the time period providing the functional constrain of the FSS algorithm [21]. This means the identification of stereotyped interictal activity. We defined in the exemplificative case a short time interval (4 ms) preceding the spike of the spike-and-wave (SW) pattern typical of the patient (Figure 1A).

FSS is a modification of ICA method considering *a priori* knowledge about the source, for example derived from the evoked potentials [23,24] or other behavior-related activities [25]. A fingerprint functional constraint is added to the cost function of the FastICA algorithm to extract the activity generated by the source of interest. In the present case the functional constraint was the maximum of the power in the short intervals identified by the spike (time 0) and included the 4 ms preceding it (from −4 to 0 ms). The lambda parameter, i.e., the balancing value between the ICA cost function (assuring the independence) and the functional constraint, was set to 1000. The optimization of the cost function was achieved by means of simulated annealing [26], starting from a temperature initialization value of 1000.

The output of the FSS algorithm is the time course of the extracted functional source (FS) activity (FS_Epi (t)) and the mixing matrix A_FS_, expressing the source topographical distribution over the scalp (Figure 1A).

In addition to FS_Epi, we applied the same constraint to the residual signal obtained by:

EEG_rp = EEG − A__Epi_ ∙ FS_Epi

converging to the source describing the remaining part of the brain network (FS_rp), similarly described by the distribution of the weights on the scalp A__rp_ and its time evolution FS_rp(t).

#### 2.1.2. Higuchi Fractal Dimension (HFD) of the Identified FS(t)

Many previous studies demonstrated that a simple method of nonlinear dynamics like Higuchi fractal dimension (HFD) [1,2,7,27,28,29,30,31] is very efficient in analysis of neurodynamics complexity [32].

In this study (Figure 1B), HFD was applied to evaluate the impact of tDCS on FS_Epi complexity and on the complementary network (FS_rp). HFD was calculated in one-second non-overlapping windows and averaged over all windows along the about 15 min of EEG recordings in each of the four conditions (see Figure 1). To select the k_max_ parameter, the HFD was calculated in the range from 1 to 100 and the value to 40 was chosen.

#### 2.1.3. Connectivity Analysis between Identified FSs(t): Directed Transfer Function (DTF)

A connectivity analysis between the FS_Epi and the residual FS_rp networks was performed by Directed Transfer Function (DTF) [33]. DTF is a directed and multivariate connectivity method defined in the frequency domain [34,35] that allows for estimation of causal influences between involved networks in a given frequency band. The multivariate approach permits for elimination of spurious connections.

To optimize the DTF calculation (Figure 1C), FS_Epi and FS_rp were down-sampled to 250 Hz and windowed in 20-s segments along the approx. 15 min of EEG recordings in each of the four conditions (see Figure 1). Then, the connectivity matrices of DTF were estimated in full frequency range up to 125 Hz.

### 2.2. Exemplificative Case—A DRE Person Who Benefitted of Single-Session tDCS

The exemplificative case comes from a study approved by the Ethics Committee of the Campus Bio-Medico University (UCBM). Informed consent was obtained from the subject involved in the study.

**Figure 2:**
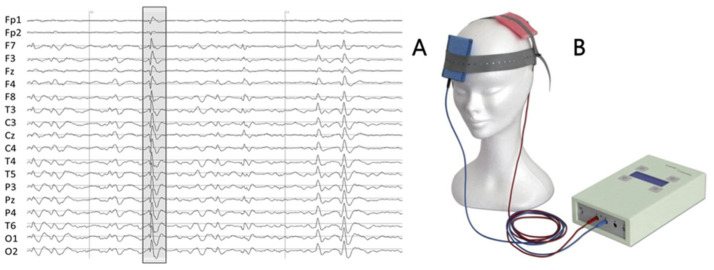
(**A**) An example of 2 s patient’s EEG, with typical interictal epileptiform discharge (IED) for this patient (irregular spike-and-wave complex) highlighted. (**B**) Battery-driven stimulator and two saline-soaked sponge electrodes.

A twenty-year old male with drug-resistant focal epilepsy participated in this study (Figure 2A). Interictal activity was mainly generated in the temporal lobe determining the tDCS cathode electrode position over T4. Clinical presentation was also compatible with a generator in the right temporal lobe. He reported an average of 100 seizures a week during the last 3 months before tDCS. The patient presented moderate cognitive impairment but had no psychogenic seizures or other major psychiatric or neurological disorders.

#### 2.2.1. tDCS Protocol

The patient underwent two double-blind stimulations (tDCS and Sham). The real stimulation (tDCS) was performed as the first one. A current of 1 mA was delivered using a battery-driven stimulator (Schneider Electronic, Gleichen, Germany-Newronika) for 20 min to two saline-soaked sponge electrodes (5 cm by 7 cm), with the cathode placed over the epileptic focus at electrode T4 and the anode placed over T3 (Figure 2B). After one month the second stimulation (Sham) was applied. After ctDCS he reported a sharp decrease in seizure frequency, with only 3 events in the week after the stimulation.

#### 2.2.2. EEG Acquisition and Preprocessing

One-hour 19-channel EEG (Figure 2A) with the sampling frequency of 1000 Hz and the reference electrode at Fcz (Micromed, Treviso, Italy) was recorded before and about four hours after each stimulation at rest with eyes closed. Off-line, independent component analysis (ICA) without epoch exclusion was applied to remove the EEG artifacts [36].

## 3. Results

### 3.1. SW Patterns for FS_Epi Identification

SW patterns were marked to identify the FS_Epi before Real stimulation, which served for the entire analysis (Figure 1).

### 3.2. Functional Source Separation (FSS)-Identified Networks

**Figure 3:**
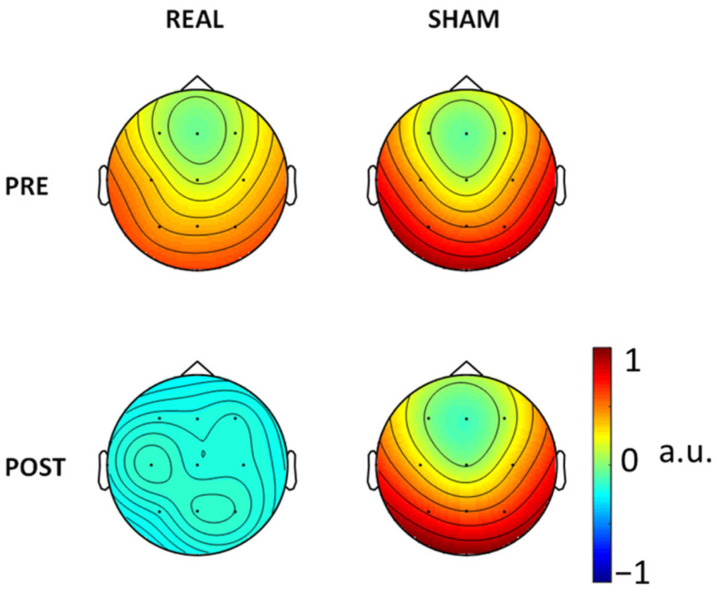
FS_Epi topographical representation (arbitrary units, a.u.) via the weights on the recording channel positions resulting from the constraint applied in the four conditions.

**Figure 4:**
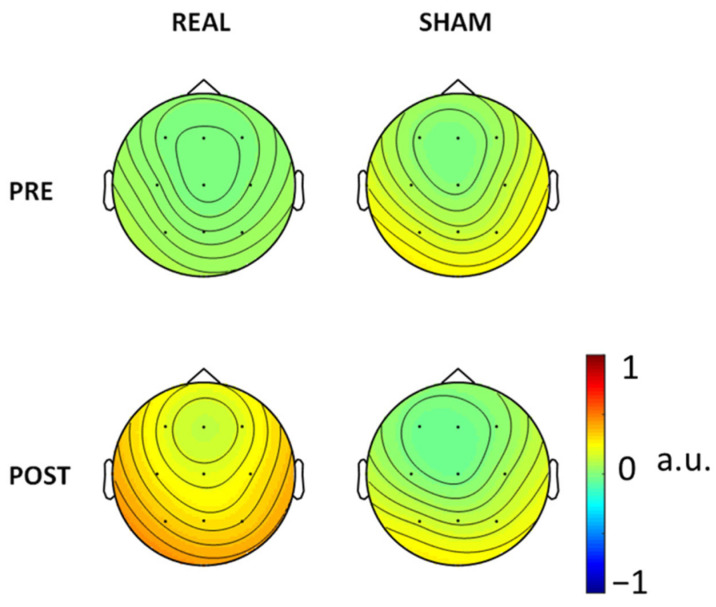
FS_rp topographical representation.

For consistency aims, we also ran the FSS algorithm in every condition, PRE and POST Real and Sham (Figure 3). There is consistency of the FSS output as derived in the three conditions (pre-Real, pre-Sham and post-Sham) while the clear reduction in SW patterns post-Real tDCS impaired the extraction in that condition. In Figure 4 we also show the FS_rp in four conditions, which were consistent across all conditions.

### 3.3. Higuchi Fractal Dimension (HFD)

**Figure 5:**
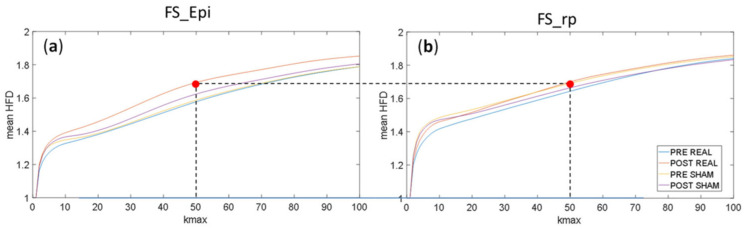
Dependence of average HFD in function of parameter k_max_ in four conditions: PRE REAL, POST REAL, PRE SHAM and POST SHAM calculated for: (**a**) epileptogenic network (FS_Epi) and (**b**) the residual network (FS_rp).

Comparison of the dependencies of average HFD, calculated for FS_Epi generated by the epileptogenic network, on parameter k_max_ (Figure 5a) showed differences between conditions. The HFD of the epileptogenic network showed an increase selectively after Real stimulation in the full range of k_max_ with respect to the other three conditions, which showed signal complexity almost stable; whereas the activity of the FS_rp residual network showed stable signal complexity across the four conditions (Figure 5b). If we look at k_max_ = 50, we see that HFD of the FS_Epi network was around 1.6 in the three conditions but after Real stimulation when it became 1.68, similar to the values displayed by the FS_rpresidual network.

### 3.4. Connectivity Analysis: Directed Transfer Function (DTF)

**Figure 6:**
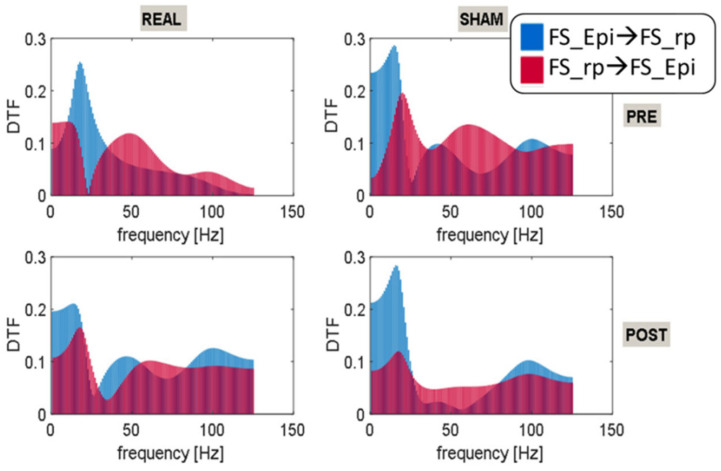
Directed Transfer Function calculated for source (marked in blue color) and for residual activity (marked in red color) in the four conditions: PRE REAL, POST REAL, PRE SHAM and POST SHAM.

The connectivity analysis performed by means of DTF showed an interesting frequency-dependent effect of stimulation. Before ctDCS, we found a 20 Hz dominance of activity flow from the source to the remaining part of brain (Figure 6). After stimulation the source oscillations were entrained in the frequencies of the residual activity, and they were shifted mainly to the lower frequencies. The spectrum profile remained similar after one month, when Sham stimulation was applied. As a result of the Sham stimulation, the beta and low gamma bands were significantly suppressed.

## 4. Discussion

The main result of our work is the design and initial validation of a pipeline for the identification of the network generating the epileptic interictal electric activity, allowing the study of its time course under different experimental conditions. The proposed FSS algorithm opens up a relevant opportunity to investigate the relationship between the epileptogenic network and the rest of the brain.

In the present proof-of-concept study performed in a single subject, we took the opportunity to evaluate the changes in the network dynamics in terms of the signal complexity when a neuromodulation session, capable of improving the weekly seizure frequency [17], intervened by modifying the network connectivity [22]. We observed that, selectively after the Real ctDCS stimulation, the epileptogenic network generated an activity with higher complexity, indicated by a higher fractal dimension and reaching the values displayed by the non-epileptogenic network (the residual FS_rp network). Fractal dimension values were lower and relatively stable in the two conditions without stimulation (pre-Real and pre-Sham) and post-Sham.

While the neurodynamics modified by neuromodulation appeared to return to baseline values after about one month, functional connectivity showed more lasting changes. In fact, the direct flow from the epileptogenic network to the rest of the brain reduced from the beta to below theta frequency ranges and was maintained after one month. In a previous study, we found that the functional connectivity changes may contribute to explain the effects of ctDCS in epilepsy, offering a new scenario in the personalization of neuromodulation interventions in people with epilepsy, as the epileptic seizure reduction correlated with the increase in the connectivity of the epileptic focus with the rest of the brain in particular in the theta band [22]. In agreement, here the directed connectivity from the epileptogenic network to the other part of the brain shifted from the beta before neuromodulation to the frequencies below theta after. After Sham stimulation, signs of reciprocal suppression appeared in the low beta and gamma drives by both FS_Epi and FS_rp, placing potential caveats on the effect of Sham neuromodulation, as is well known with placebos from the experience of pharmacological clinical trials.

The present approach can be a valid tool to enhances the understanding of the relationship between neurodynamics characteristics and the symptoms suffered by people with epilepsy. In fact, the investigation of neuronal network characteristics and dysfunction underlying interictal epileptiform discharges will enhance understanding of seizures’ generation [37,38,39].

Highlighting future research directions, further knowledge on the neurodynamics of epileptogenic networks is proposed with a view to improving the interaction through neuromodulation with epileptogenic networks [3]. In addition, interesting differences in the duration of neuromodulation-induced changes in neurodynamic characteristics and functional connectivity between epileptogenic networks and the rest of the brain will be the subject of future investigations.

Present data strengthen previous literature about the role of reciprocal competition of epileptogenic and the other brain networks in determining the balance between seizures and neurological performance in people with epilepsy. Even if as a proof-of-concept study, this manuscript offers reliable scientific fundaments to the study of pathophysiological neural pathways sustaining the strength of the epileptogenic networks, also demonstrating its sensitivity to external neuromodulating intervention. These sustain the strenuous efforts of the neurophysiological scientific community in testing new neuromodulation therapies to fight epilepsy.

## Data Availability

The data presented in this study are available on request from G.A.
